# Unveiling the unknown phylogenetic position of the scallop *Austrochlamys natans* and its implications for marine stewardship in the Magallanes Province

**DOI:** 10.1038/s41598-021-86492-9

**Published:** 2021-03-31

**Authors:** Sebastián Rosenfeld, Cristian Aldea, Zambra López, Claudia S. Maturana, Jaime Ojeda, Francisco Bahamonde, Camille Detrée, Andrés Mansilla, Elie Poulin, Karin Gérard

**Affiliations:** 1 Laboratorio de Ecología Molecular, Departamento de Ciencias Ecológicas, Facultad de Ciencias, Universidad de Chile, Las Palmeras # 3425, Ñuñoa, Santiago Chile; 2 Laboratorio de Ecosistemas Marinos Antárticos Y Subantárticos, Universidad de Magallanes, Avenida Bulnes 01890, Punta Arenas, Chile; 3 Instituto de Ecología Y Biodiversidad, Las Palmeras 3425, Ñuñoa, Santiago Chile; 4 Centro de Investigación Gaia-Antártica, Universidad de Magallanes, Avenida Bulnes 01855, Punta Arenas, Chile; 5 Research Center Dynamics of High Latitude Marine, Ecosystem (Fondap-IDEAL), Universidad Austral de Chile, Casilla # 567, Valdivia, Chile; 6 School of Environmental Studies, University of Victoria, 3800 Finnerty Road, Victoria, BC Canada

**Keywords:** Phylogenetics, Evolution, Taxonomy, Ecology, Biodiversity

## Abstract

Two species of scallop, *Austrochlamys natans* (“*Ostión del Sur*”) and *Zygochlamys patagonica* (“*Ostión patagonico*”) are presently exploited in the southern part of the Magallanes Province (MP). The lack of clarity in taxonomic identification and ecological aspects is generating both erroneous extraction statistics and an unperceived harvesting pressure on *A. natans* and *Z. patagonica*. We aim to discriminate these Magallanes scallops accurately, improve our understanding of their complex natural history and discuss possible implications for their management and conservation status, given the current fisheries statistics. To achieve these goals, we present a complete review of the historical identification of the Magallanes scallop and a multi-locus molecular phylogeny which allowed us to recover the phylogenetic position of *A. natans*. We sampled 54 individuals from five localities across the southern Pacific coast of the MP. We calculated the depth of the byssal notch (BND) and shell height (VH) ratio from morphological characters and conducted phylogenetic reconstructions with mitochondrial (12S and 16S) and nuclear markers (28S) using Bayesian and maximum likelihood analyses. Both morphology and molecular phylogeny identified two distinct entities, *Z. patagonica* and a distinct, highly divergent lineage that corresponds to *A. natans*. Our study provides integrative evidence to alert the current fishery management and the need for further conservation studies.

## Introduction

The Pectinidae (scallops) are one of the most morphologically, behaviourally and biologically diverse families in the class Bivalvia^1^. Pectinids are an important component of many benthic communities; most species are found in the shallow waters of the continental shelves, with a very few species restricted to deep and abyssal waters down to 7000 m depth^1^. In addition to their ecological relevance, scallops are an important economic resource taxon that supports many artisanal and industrial fisheries worldwide^2,3^. However, despite their ecological and economic importance, the taxonomy and systematics of pectinids are still in development^4^. The members of the family Pectinidae can be identified by two key structures: (1) a particular hinge-ligament system that connects the two valves of the shell and (2) the presence of a ctenolium^1^. However, the taxa within the family Pectinidae exhibit a wide range of modifications of the general phenotype, with striking shape, texture, and colour variants of the shell^1^. This high morphological variability has caused some classification problems, mainly because the taxonomy of the family is generally based on macroscopic characters and the shell form of adults^1,5^. For example, one of the first classifications of scallops was mainly based on the thickness, coloration, and degree of exterior sculpture of the valves^1^. Nevertheless, the high morphological plasticity and convergent evolution of the bivalve shell casts some doubt on the validity of classification schemes^1^. In addition, due to the homoplasy of several morphological characters, it has been observed that distant species within the phylogeny present some similar morphological characters^1^. This makes the distinction of sympatric species difficult due to the lack of clear diagnostic characters^1^. Consequently, Serb^1^ concluded that, as shell shape evolved in a repetitive fashion it does not express high-level taxa, but rather reflects life habits^6–8^. Raines and Poppe^9^ made a full monograph of the family Pectinidae, recognizing 250 species. So far, the most used and accepted classification is that of Waller^5^, which divides the Pectinidae into four subfamilies and 10 tribes. Although Waller^5^ does not include molecular data, this classification is consistent with the most comprehensive molecular phylogeny constructed to date^7^, except for the position of the tribe Aequipectini^1,3^. The use of molecular tools over the last two decades has contributed to a better understanding of the taxonomic status and phylogenetic relationships among scallops around the world^1,5,7,10,11^.

Six scallop species occur along the Chilean coast: *Argopecten purpuratus* (Lamarck, 1819), *Delectopecten polyleptus* (Dall, 1908), *D. vitreus* (Gmelin, 1791), *Zygochlamys phalara* (Roth, 1975), *Z. patagonica* (King, 1831) and *Austrochlamys natans* (Philippi, 1845). Only *Ar. purpuratus* (northern Chile), *A. natans* and *Z. patagonica* (both southern Chile) have been exploited commercially^12^. Both *A. natans* and *Z. patagonica* are distributed from the Chiloé Archipelago (42°S) to Cape Horn (55°S)^13^, but only *Z. patagonica* extends its distribution along the Atlantic coast up to Uruguay^14^. *Z. patagonica* is currently part of the tribe Chlamydini (subfamily Chlamydinae); *Austrochlamys natans* belongs to the monospecific tribe Austrochlamydini^13^. This latter tribe is missing in Wallers’s^5^ classification, however Raines and Poppe^9^ included it in the subfamily Chlamydinae without description or illustration. Subsequently, Dijkstra^15^, after a complete taxonomic revision of Pectinidae, incorporated the Austrochlamydini in the Pectininae. Despite the great contributions of Waller^5^, Raines and Poppe^9^ and Dijkstra^15^, pectinid classification has been modified by recent molecular-based phylogenetic studies^4,7^. One of the great challenges within the Pectindae is to be able to include more new taxa in the phylogenies, mainly to fill the gaps in the morphology-based classification systems^1^.

The distinction between *A. natans* and *Z. patagonica* has been complex historically in the Magallanes Province (MP), due to the high morphological similarity and lack of clear diagnostic characters^16^. Indeed, several authors^16–18^ mentioned the presence of two commercial pectinids in the MP, but without clear identification criteria^16^. Waloszek^19,20^ performed a comparative morphological study of the two species, concluding that they differ in spermatozoid head shape, shell texture and growth parameters, however, conclusive evidence was not provided mainly because of the overlapping of morphological characters^16^. This lack of clarity is generating a complex scenario in which the characters traditionally used would not be discriminating between the species; *A. natans* has not been included in any current phylogeny^3,4,7,11^ so its phylogenetic position is unknown.

The Chilean fishery statistics erroneously attributed the entire scallop resource to *Z. patagonica* during the 1980s, when in reality *A. natans* was also harvested^16^. Valladares and Stoltz^16^ conducted a study of the growth of *Z. patagonica* to discriminate these species, concluding that its maximum length would not reach 75 mm, while *A. natans* can exceed 100 mm. Consequently, in the 1990s the Chilean National Fishing Service (SERNAPESCA) established a criterion for the scallop fishery based on the maximum length and established the minimum catch size as 75 and 55 mm, respectively for *A. natans* and *Z. patagonica*. Since then the scallop fishery has been based mainly on the extraction of *A. natans* (see Supplementary Fig. S1). Moreover, since *Z. patagonica* would not reach relevant commercial size, since 2013 SERNAPESCA has considered the scallop fishery as a monospecific resource (see Supplementary Fig. S1). However, other technical and fishery reports have indicated sizes greater than 75 mm for *Z. patagonica*^13,21^, and an average size under 75 mm in natural “banks” of *A. natans*^22^. Consequently, stakeholders of the fishing industry and artisanal fishermen have suggested reducing the minimum catch size ^23^. All this historical background indicates a complex scenario in which banks of *Z. patagonica* could be considered as banks of small *A. natans* and fisheries reports are considering mixed banks as monospecific. The lack of taxonomic characters discriminating the species is leading to erroneous extraction statistics of *A. natans* and an unperceived harvesting pressure for both species. Therefore it is very relevant to be able to identify the two species correctly and to know the phylogenetic position of *A. natans* in the context of the regional fisheries and future conservation strategies.

Our study has two main objectives: (1) elucidate the phylogenetic position of *A. natans* and (2) distinguish accurately the Magallanes scallops *Z. patagonica* and *A. natans* in order to improve our understanding of their complex natural history and fisheries statistics significantly. For this, we first defined the phylogenetic position of the Magallanes scallops among 90 species of Pectinidae and discussed their respective taxonomy, allowing us to test whether the hypothesis of Dijkstra^15^ or that of Raines and Poppes^9^ is verified: i.e. if the monospecific tribe Austrochlamydini belongs to the subfamily Pectininae or the Chlamydinae. Some aspects of the biogeography of scallops in the Southern Ocean are also discussed in light of these new results. Second, we conducted an exhaustive review of the morphological characters of each species, then evaluated them using the morphological criteria established by Jonkers^13^ and sequences of ribosomal markers. Finally, we discuss how phylogenetic and taxonomic information will have important implications for fishery and conservation management of *A. natans* and *Z. patagonica* in the MP.

## Results

### Identification and morphological analyses

**Figure 1:**
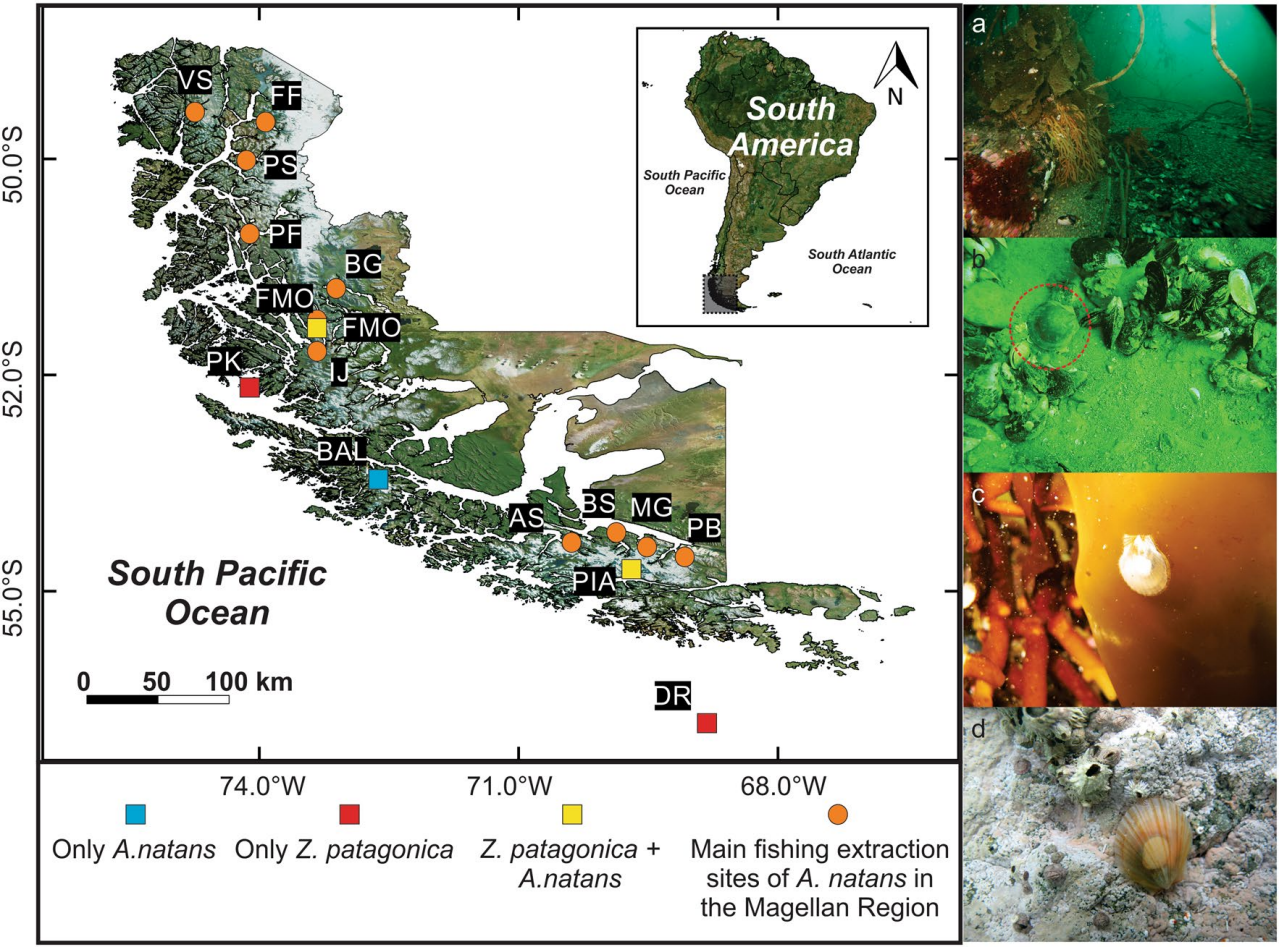
Sampling locations in the Magallanes Region in squares. From north to south: FMO: *Montaña* Fjord; PK: Parker Island; BAL: *Ballena* Fjord; PIA: Pia Fjord-Beagle Channel and DR: Diego Ramirez Archipelago. Main fishing extraction sites of *A. natans* in the Magellanic in orange circles. From north to south (extracted from Comité Científico Bentonico 2018): VS: Ventisquero Sound; FF: Falcon Fjord; PS: Penguin Sound; PF: Peel Fjord; BG: Balmaceda Glacier; FMO: *Montaña* Fjord; IJ: Jaime Island; BAL: *Ballena* Fjord; AS: Agostini Sound; BS: Brook Sound; MG: Marinelli; PB: Parry Bay. Columns of photographs show different habitats of collected samples: (**a**) typical habitat of specimen collected in this study, (**b**) Adult scallop on muddy bottom in FMO, (**c**) Juvenile scallop attached to a kelp (**d**) Juvenile scallop on calcareous algae bottom. The map was created in QGIS software ver. 3.16^46^ available in: https://qgis.org/en/site/. *Credits to photographs Jaime Ojeda*.

**Figure 2:**
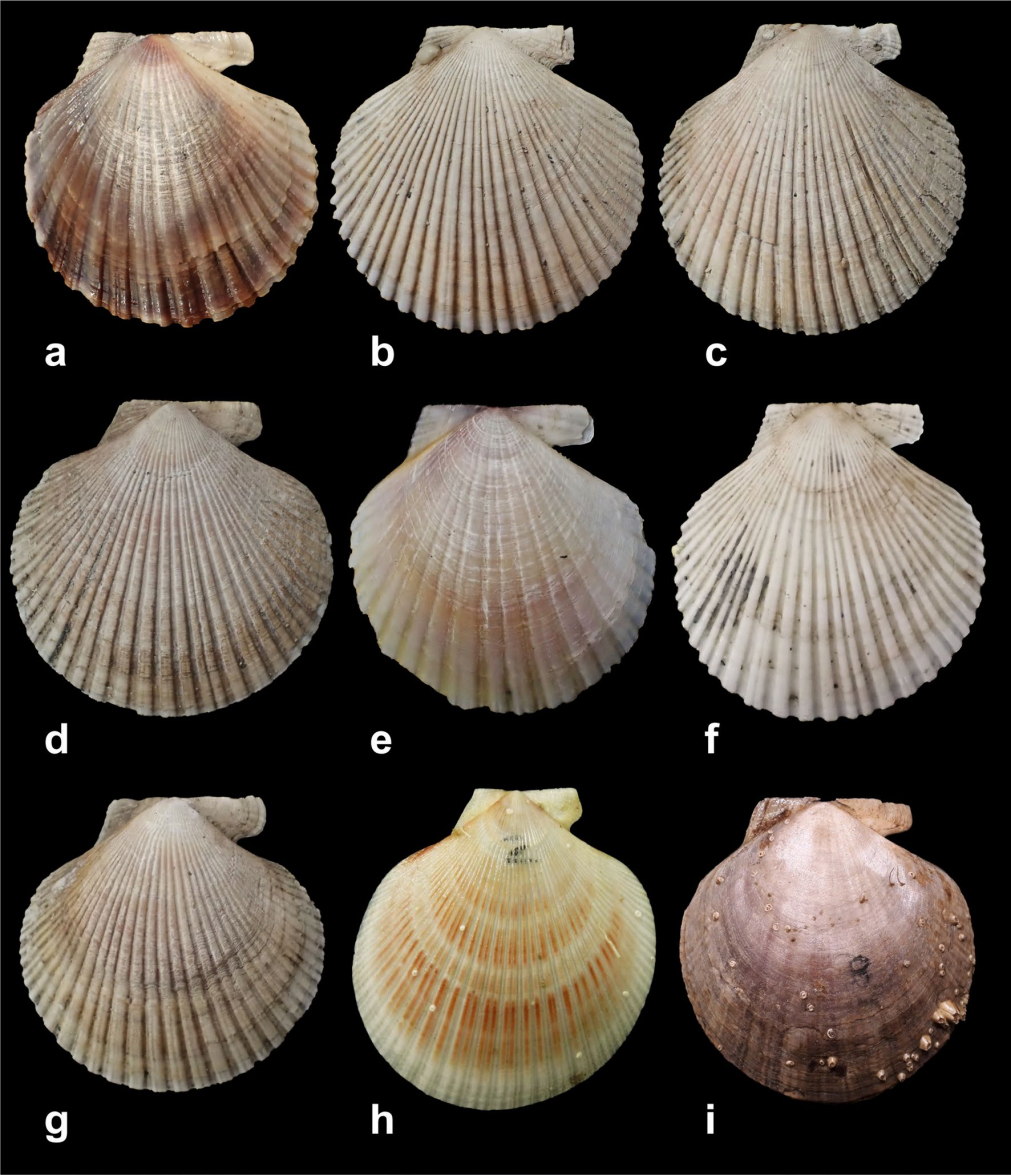
Photographs of Magallanes scallops. (**a**) and (**e**) *Austrochlamys natans* from Pia Sound (**b**–**d)**, (**f)**, (**g**) *Zygochlamys patagonica* (Pia Fjord), (**h**) *Z. patagonica* (Diego Ramirez Archipielago), and (**i**) *A. natans* (Montaña Fjord)*.*

The individuals identified in the five locations (Fig. 1) were mostly adult specimens over 40 mm in length, except for individuals from Parker Island that ranged between 20 and 38 mm length. The individuals identified morphologically as *Austrochlamys natans* presented a moderately long shell, with an average shell height (VH) of 77 ± 9.75 mm. The shell coloration was quite variable, from white to dull brown or purple-brown to purple (Fig. 2a,e,i). The individuals identified as *Zygochlamys patagonica* presented a medium-sized shell with an average shell height (VH) of 41 ± 6.59 mm. The shell coloration was mainly white or pale and brick-red (Fig. 2b,c,d,f,h).

**Figure 3:**
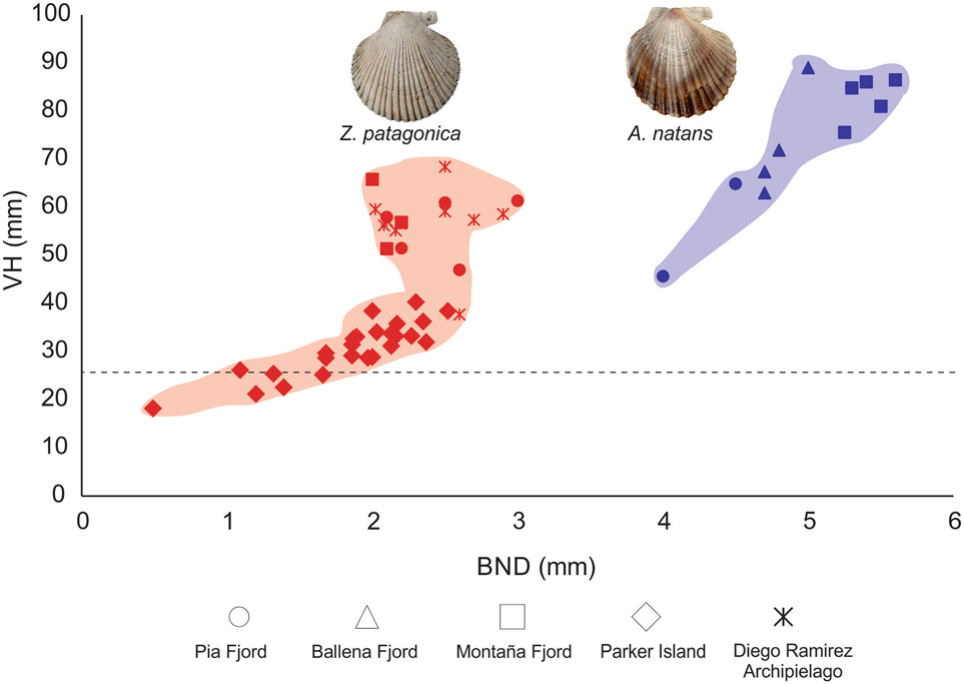
Bivariate plot of depth of byssal notch (BND) against valve height (VH) of individuals of *Zygochlamys patagonica* (in red) and *Austrochlamys natans* (in blue) collected in the MP.

**Figure 4:**
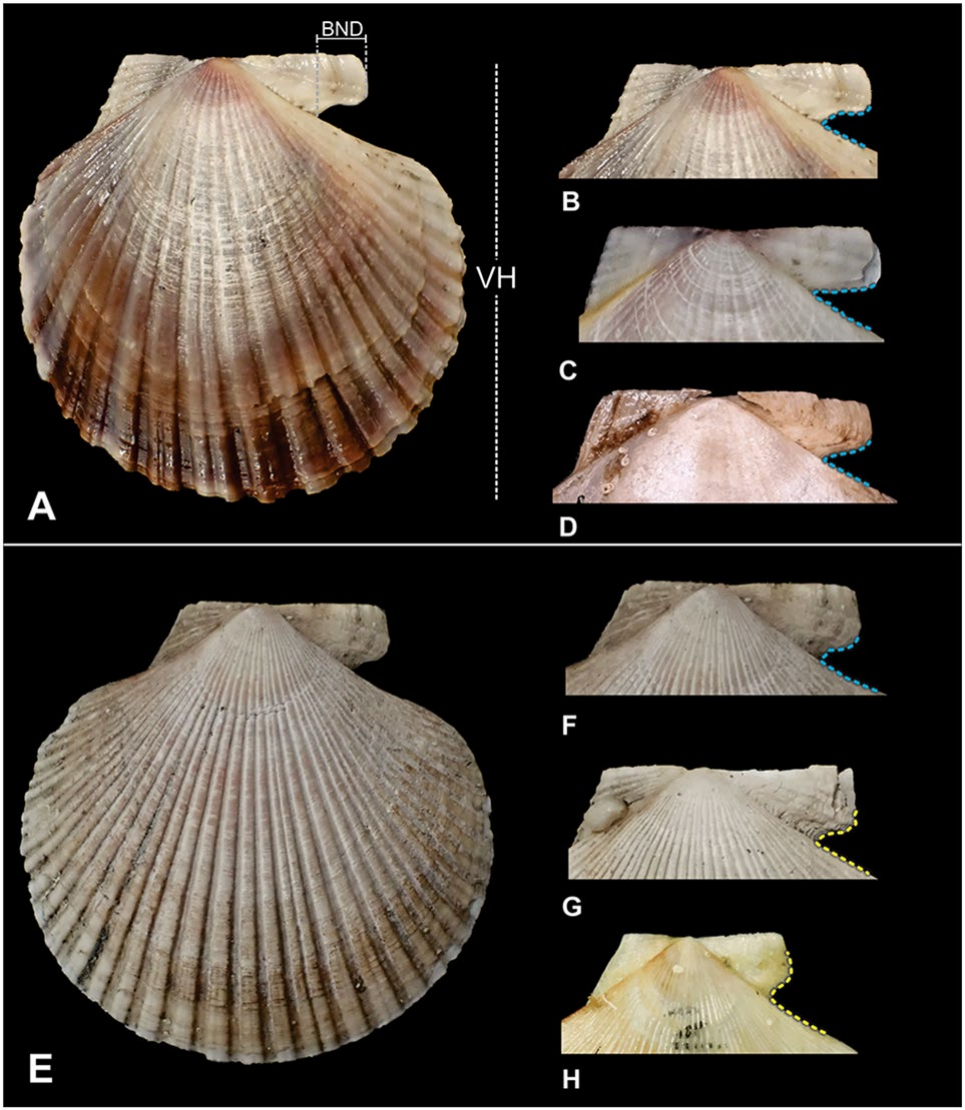
Linear measurements used in this study for the individuals of *A. natan*s and *Z. patagonica*. (**A**–**D**) *A. natans* and (**E**–**H**) *Z. patagonica*. VH = valve heights, BND = deep of the byssal notch. The dotted lines highlight the outline of the byssal notch, emphasizing whether it is acute (light blue) or arcuate (yellow).

Individuals of *A. natans* presented a greater depth of the byssal notch (BND), with an average depth of 5.60 ± 0.74 mm (Fig. 3) and an arcuate (Fig. 4B) to acute byssal notch (BN) (Fig. 4C,D). The individuals of *Z. patagonica* presented a lower BND (Fig. 3) and a more arcuate BN (Fig. 4F–H). The analysis of the BND/VH relationship discriminates two very different groups (Fig. 3), one composed of all specimens of *Z. patagonica* (red color), and the second with all specimens of *A. natans* (blue color). Individuals of *Z. patagonica* had an average BND/VH ratio of 0.05 ± 0.01, while those of *A. natans* had an average BND/VH ratio of 0.08 ± 0.01. This difference between the species was statistically significant (U = 2, *p value* = 0.0000).

### Phylogenetic analysis of pectinids

The length of fragments amplified in *Zygochlamys patagonica* and *Austrochlamys natans* for the 12S, 16S and 28S loci are indicated in Table S2. The levels of interspecific divergence (p-distance) between haplotypes of the species were rather high: 5.3% (28S), 13.1% (16S) and 18.7% (12S), reaching 10.8% for the concatenated sequences. The sequences of the specimens previously identified as *Z*. *patagonica* by Jonkers’ (2003) method resulted highly similar to the sequences of *Zygochlamys patagonica* in Balmaceda Glacier (BG). All samples identified by Jonkers measurement as *A. natans* shared the same haplotype, new to Genbank and to scallop genetic diversity. No intraspecific variation was observed for either species at any locus in the Magallanes Region (between 50 and 56°S).

Due to the appearance of insertions/deletions and gaps during the alignment with the other 90 pectinid sequences and the 2 propeamussiids, the datasets consisted of 338, 492, 899 base pairs (bp) for 12S, 16S and 28S, respectively. Some ambiguously aligned hypervariable portions were removed from the 16S dataset. Single-locus trees (Figure S3,S4,S5) were congruent and mainly differ in the low support of some nodes, as the 3 loci have their own resolutive power at different depth of the tree. The concatenation allowed a more substantial node resolution in the multi-loci tree. The concatenated sequence set had 1659 bp, 726 polymorphic sites and 550 parsimoniously informative sites. No substitution saturation was detected in the combined dataset.

**Figure 5:**
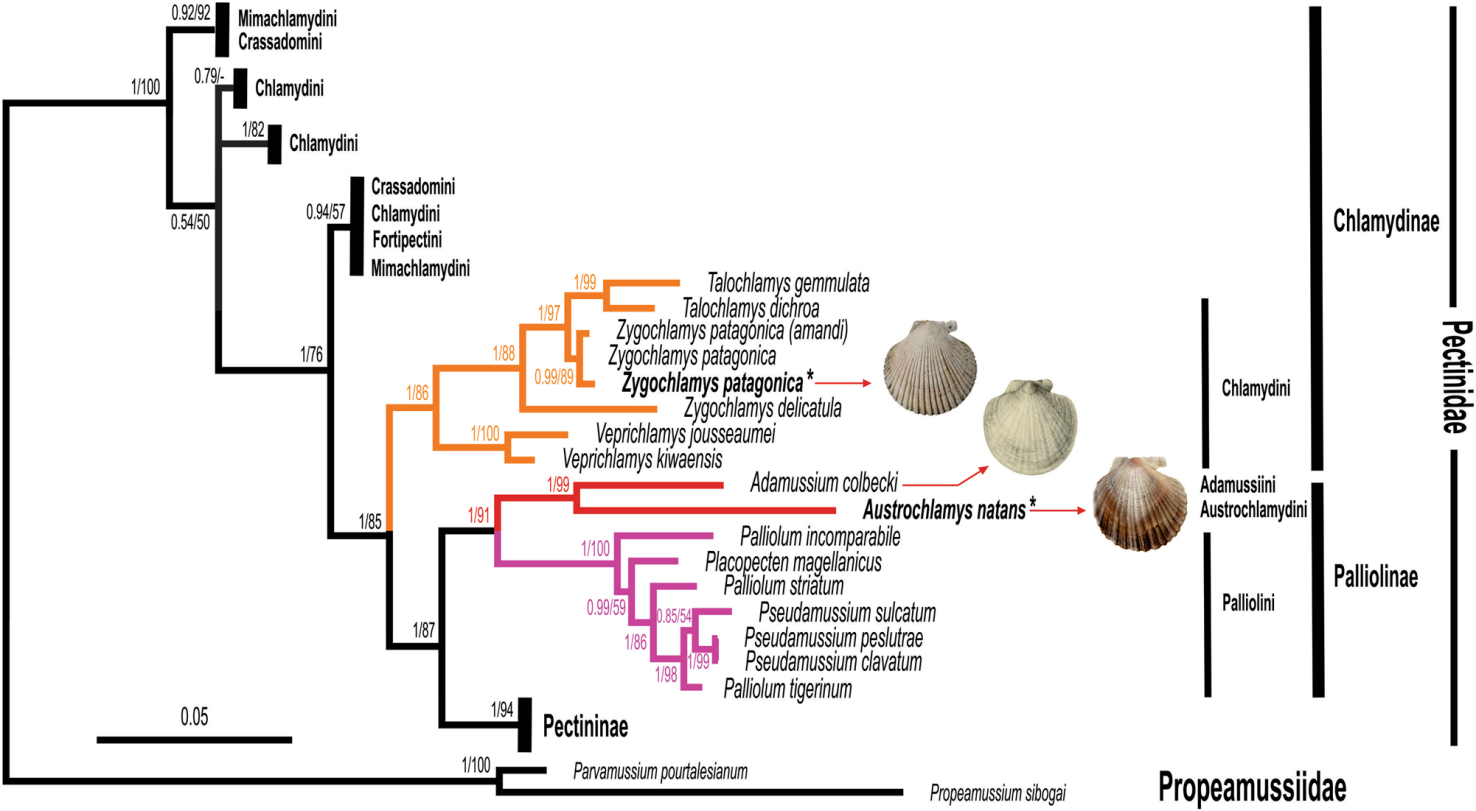
Phylogenetic relationships among 92 pectinid species based upon the combined dataset (12S-16S-28S) using Bayesian inference (BI) and maximum likelihood (ML, not shown) probabilistic methods. Branch support values are indicated near each node: The first number represents the portion of sampled trees in which the node was found (posterior probability, pp); the second number is the bootstrap support (bs) value > 50; (−) indicates values < 50. The illustration of *Ad. colbecki* was modified from Smith^24^.

The species included in the family Pectinidae cluster in a single clade, suggesting the monophyly of the family. This clade has full support in phylogenies reconstructed by both approaches. BI and ML gave congruent topologies, however ML generally gave lower bootstrap support (bs) than BI posterior probabilities (pp). The base of the pectinid tree is rather four-fold. Only the Chlamydinae appear paraphyletic among subfamilies; Palliolinae and Pectininae are monophyletic and highly supported (Fig. 5). Lineage H is a highly supported monophyletic group, composed of species of the tribe Chlamydini (*Veprichlamys* spp., *Zygochlamys* spp., *Talochlamys gemmulata*, *T. dichroa*) which is sister to the clade grouping Palliolinae-Pectininae, also very well supported.

The Magallanes samples of *Z. patagonica* were tightly associated with its conspecific in a clade (pp: 0.99; bs: 89). The Magallanes specimen of *Austrochlamys natans* (Austrochlamydini) clustered with *Adamussium colbecki* (Adamussini) in a fully supported clade. Both monospecific tribes were nested with the tribe Palliolini in the subfamily Palliolinae (Fig. 5).

## Discussion

This is the first comparative study of commercial scallop species in the Pacific coast of the MP combining morphological and molecular characters. Our phylogenetic analyses highlight the association between *A. natans* and *Ad. colbecki*; two members of monospecific tribes and last extant representatives of their Southern Ocean-restricted genera.

These results confirm the presence of both Magallanes scallops in the MP, as well as the so-far unsuspected presence of mixed "banks" where both species occur in sympatry. The BND/VH ratio helps discriminate between two distinct entities that belong to the genetic lineage of *Z. patagonica* and to a different lineage, highly divergent from the former, which corresponds to *A. natans*. *A. natans* is the only species of a whole lineage with a particular phylogenetic value, therefore having developed and tested an accurate identification criterion for both scallops will allow efficient fishery management in the future.

Here we discuss the phylogenetic position and the taxonomic status of both Magallanes scallops, as well as the implications of these results for the future management and conservation of *Z. patagonica* and *A. natans* in the Magallanes Region. Despite the numerous classifications built on morphological, ecological or molecular data, the relationships among pectinids are still under constant modification depending on the number of taxa, loci, length of the sequence and the selected outgroups^1,4^. The work of Alejandrino et al*.*^7^ is the most inclusive so far in terms of taxon sampling, with 81 species. Although Scherrat et al*.*^25^ included 143 species, the node supports of the phylogenetic trees are not provided, making it difficult to assess the robustness of this large phylogeny. In order to define the phylogenetic position of *Zygochlamys patagonica* and *Austrochlamys natans*, we included 93 pectinid taxa (43 genera) representative of tribes Chlamydini, Crassadomini, Fortipectini, Palliolini, Aequipectinini, Pectinini and Amussini. Comparing to Waller's^5^ and Dijkstra’s^15^ classifications, only the subfamily Camptonectinae and the tribe Mesoplepini are missing. We used three ribosomal regions (one nuclear and two mitochondrial). Compared to Alejandrino et al*.*^7^, histone H3 is missing here, however this locus is among the least informative^4^. The family Pectinidae appears to be monophyletic with high support values (Fig. 5, S2), as previously demonstrated^4,7,26–28^. According to Dijkstra^15^ there are currently five subfamilies of Pectinidae, two of which are absent from our analysis: Camptonectinae and Pedinae. This topology supports the classifications of Waller^5^ and Dijkstra^15^, except for the position of the tribe Austrochlamydini.

Our Magallanes scallops separated into two very divergent clades: *Z. patagonica* is associated with its conspecifics and congenerics in a single lineage (Fig. 5), which also contains species of *Veprichlamys* and *Talochlamys*. This lineage already appeared well supported as the sister clade to Palliolinae and Pectininae in Alejandrino^7^. For the first time, *Talochlamys dichroa* and *T. gemmulata* are nested with high support values into the *Zygochlamys* clade, making this latter genus paraphyletic (Fig. 5). These taxa are all restricted to high latitudes of the Southern Ocean. Due to phylogenetic and geographic affinities, we suggest that these three genera may constitute a tribe separate from Chlamydini. Since Dijkstra^15^ moved the two Atlantic ‘*Crassadoma’* into the genus *Talochlamys,* the affinities among *Talochlamys* spp. had not been explored until now. *Talochlamys* species rather associate according to geographic affinities, splitting the genus into two highly divergent entities corresponding to European and New Zealand *Talochlamys*. A systematic revision of these four species would be useful.

*Austrochlamys natans* associated with the Palliolinae, which was elevated to a subfamily rank by Waller^5^. Of the three extant tribes that compose this group, Mesopleplini are missing from our phylogenetic analyses. We included 4 genera (8 species) of the remaining two tribes: *Adamussium* (Adamussini) and *Palliolum*, *Pseudamussium*, *Placopecten* (Palliolini). The present sampling of Palliolini is the most inclusive to date and led to the monophyly and full support of the tribe Palliolini. Our phylogenetic results do not support any of the previous classifications of the tribe Austrochlamydini^1,5,9,13,15^, and introduce this monospecific tribe as a new member of the subfamily Palliolinae. Indeed, *Austrochlamys natans* clusters together with *Adamussium colbecki,* both in a sister clade to Palliolini. The first molecular characterization of *Ad. colbecki* did not lead to a clear classification due to the low polymorphism of the 18S^26^. Later, *Ad. colbecki* appears either as sister species to Chlamydinae or to Palliolini, depending on tribe sampling and the choice of outgroup and loci^4,10,11^. However, in the most recent and inclusive studies of taxon sampling^7^ (present study) or genomic cover^29^, *Ad. colbecki* is the sister group of the tribe Palliolini, as in the present phylogeny.

The subfamily Palliolinae originated from a Chlamydinine ancestor in the Cretaceous and subsequently underwent diversification in the Northern Hemisphere^1^ and in the Southern Hemisphere, where the extinct genus *Lentipecten* spread in the Paleocene–Eocene Thermal Maximum^30^. The genus *Adamussium* derived from *Lentipecten* and appeared in the early Oligocene; it comprises 5 endemic Antarctic species; *Ad. colbecki* is the only one extant^13,31,32^. The genus *Austrochlamys* also appeared in the Oligocene and was first restricted to King George Island (South Shetlands), then spread around the north of the Antarctic Peninsula and achieved a circum-Antarctic distribution until the Pliocene^13,33,34^. *Austrochlamys* persisted during the progressive cooling of the Antarctic Continent from the Paleocene to the Pliocene, dominating the coastal areas, while *Adamussium* occupied the deep seas and continental platform^33^. The opening and deepening of the Drake Passage and the intensification of the Antarctic Circumpolar Current during the Pliocene provoked a drastic cooling and the extension of sea ice over the coastal habitat, which caused the northward movement of *Austrochlamys* and its subsequent disappearance from Antarctica, along with the circumpolar expansion of *Ad. colbecki* in Antarctic shallow waters^33^. The colonization of the coastal habitat has been related to the sea ice extent that provided a more stable environment and low-energy fine-grained sediment with which *Adamussium* was associated in the deep waters. *Austrochlamys* fossils appear in the Subantarctic Heard Island in late Pliocene layers (3.62–2.5 Ma^35^). Today *Ad. colbecki* is a circum-Antarctic and eurybathic species that reaches high local density in protected locations^13,36^, while all *Austrochlamys* became extinct except for *A. natans*, which is restricted to southern South America^33^. The phylogenetic affinity highlighted here between *A. natans* and *Ad. colbecki* has its origins in the Southern Ocean; the deep divergence between the lineages of these monospecific tribes attests to the long time since their common origin in the Paleogene. These results point out both species as relevant biogeographic models to address longstanding questions regarding the origin of marine biota from Southern Ocean.

The nomenclature, taxonomy and ecology of both *A. natans* and *Z. patagonica* have been problematic for almost 200 years. Since its original description^37^, *Z. patagonica*, a.k.a. the “*Ostión Patagónico*” has been named with more than 10 synonyms, probably due to the great intra-specific morphological variability throughout its distribution^19,38^ (see the nomenclatural history in Supplementary Table S1). In contrast, there are very few records in the scientific literature and no genetic data on *A. natans,* a.k.a. the “*Ostión del Sur*”^13,14,17,19^, and some problems of nomenclature and establishing diagnostic characters persist since its description^13,39^. Many of the current junior synonyms of both species were described from small and juvenile specimens (under 52 mm VH^39–41^). Indeed, all deposited type material of *A. natans* ranges from 23.5 to 52 mm VH; the latter is half of the maximum size^39^. The criteria most commonly used for the identification of both scallops were number of radial primary ribs, maximum size, shell colour and presence of laminated concentric lines (Supplementary Table S1). Specimens with marked primary and secondary radial ribs alternated regularly and more whitish colouring of the right shell were attributed to *Z. patagonica*, while those with weaker and less markedly coloured radial ribs and the maximum size were considered as *A. natans*^42^. However, the number of radial ribs overlaps between *Z. patagonica* (26–42^12,43^) and *A. natans* (22–50^17,19^). These characters also have high variability across different environments and during ontogeny^13,17^. Thus the use of a taxonomy based on environment-sensitive and allometric characters has led to confusion in the morphological identification of these species^13,38^. The criterion used in the present study, the BND/VH ratio established by Jonkers^13^, discriminates the species efficiently. As attested by the narrow dispersal cluster in Fig. 3, this character has low intra-population variability^13^. In some cases a level of intraspecific variation can be detected, and this is mainly due to the environments where the scallop populations inhabit^19^ (e.g. exposed, protected, substrate type, fjord, oceanic). However, although there may be some intraspecific variability between populations, this variability does not generate problems for the identification of the two species. Individuals of *A. natans* generally presented a significantly greater BND/VH ratio than those of *Z. patagonica*. However, it is important to consider that, given that this character varies during ontogeny, it is more accurate in individuals over 25 mm VH^13^. Only the molecular identification was able to discriminate juvenile scallops of both species accurately.

According to the literature, *A. natans* is restricted to interior waters of channels and is associated with kelp forests of *M. pyrifera* (Supplementary Table S1). *Z. patagonica* inhabits a wider range of environments such as bottoms of shells, sand, mud and gravel in protected and exposed areas, between 2 and 300 m depth (Supplementary Table S1), but is also associated with kelp forests in fjords with different degrees of glacial retreat^12,16,44^. The juveniles of both scallops recruit in kelp forests^44,45^. According to the local artisanal fishermen, adults of "*Ostión del Sur*" (*A. natans*) occur in fjords with glaciers (orange circles in Fig. 1^23^). We included two sampling locations near glaciers (in Pia and *Montañas* fjords), where large individuals (between 46 and 86 mm) of *A. natans* and *Z. patagonica* occur in sympatry. This sympatry was previously reported in Silva Palma Fjord between 5 and 25 m depth^16^. In conclusion, scallop banks are not monospecific but rather mixed and *Z. patagonica* occurs in the interior waters of the channels and fjords. Consequently, these two species have overlapping ecology (recruiting zone and glacial affinity) in the channels and fjords, overturning a long-held view that these scallops have marked habitat segregation.

The fishery for both species was established in the 1990s in the political-administrative Region of Magallanes^16^, despite the complexity of the morphological recognition of scallops. The distinction between species was based on shell colour and radial ribs^42^, two characters that, given the results of this study, do not have this diagnostic capacity. Consequently, the scallop fisheries in the Magallanes Region are currently based on inaccurately discriminative characters. Scallop banks in MP have always been considered as monospecific^16,47^. A great part of scallop landing has always been attributed to *A. natans*^47^, about which the scientific literature is scarce (Supplementary Table S1). Conversely, *Z. patagonica*, which was erroneously considered as the commercial species of southern Chile, has more scientific research (Supplementary Table S1).

The difficulty to discriminate *A. natans* and *Z. patagonica* morphologically may lead to incorrect fishery statistics and uncertain conservation status of *A. natans*. Incorrect fishery statistics could overestimate the abundance of banks of *A. natans* compared to *Z. patagonica*. If the minimum catch size is reduced^23^ in the context of the fishing overuse of the last decade, *A. natans* may suffer a reduction of its maximum size^48^. Therefore, an identification criterion between species is a need to improve fishery management. We showcased a quantified criterion that is useful to identify both species. In the short-term, this method can be used, but it is difficult to enforce in practical ways. We suggest to train fishing inspectors, following three guidelines. First, the identification should consider only the right valve (RV) for species identification, since the left valve is not taxonomically informative. Second, for visual classification, check the outline of the BN, mainly because the individuals of *Z. patagonica* have a more arcute BN. Third, a reliable identification has to measure the depth of the byssal notch (BND) and shell height (VH) ratio. Lastly, future research and fishery monitoring should follow these criteria to carry out a correct identification and subsequently better landings statistics.

Molecular tools allowed evaluating the phylogenetic relationships of scallops globally or regionally and incorporating parameters that can be used for the management and conservation of species of commercial interest^49^. For example, in the last few decades metrics have been developed to address conservation problems that give us a measure of the current state of particular taxa. These conservation priorities are often seen as measures for threatened species categorized by the IUCN Red List (World Conservation Union, 1980), one of the most widely and recognized systems. Although this prioritization metric incorporates phylogenetic distinctiveness (PD), this factor has been updated due to the importance of quantifying the loss of evolutionary diversity that would be implied by the extinction of a species^50^. The magnitude of the PD loss from any species will depend (but not exclusively) on the fate of its close relatives^51^. The “*Ostión del Sur*”, *Austrochlamys natans* is the last representative of its tribe (Austrochlamydini) in the Southern Ocean. Its phylogenetic position and the long branch length (i.e. the length of the branch from the tip to where it joins the tree), which represents an important amount of evolutionary change, highlights the degree of isolation of *A. natans* and calls attention to the possible loss of a unique genetic lineage*.* There is currently no conservation value for this relict species; we sought to alert the current fishery management that the “*Ostión del Sur*” is a distinct taxon and provide integrative evidence for further conservation studies.

Finally, regarding the overlapping niche of these scallops and the conservation importance of the clade of *A. natans*, we propose three key recommendations for the future scallop fishery policies in the sub-Antarctic channels. First, it is necessary to assess the proportion of both species per bank and landing to generate a distribution map through the sub-Antarctic channels. For this assessment, the byssal notch depth is the most appropriate morphological character. Second, we recommend reassessments of biological and ecological parameters (e.g. size at first maturity) for *A. natans* across the glacial fjords, which are the most relevant fishing sites. As a final point, today there is a complete lack of knowledge of the genetic connectivity along the Subantarctic Channels. Thus we should generate more research about spatial population genetics at different temporal scales. The integration of genomic approaches (e.g. SNPs) with macro- and micro-environmental modelling approaches provide enormous opportunity to establish a new regional zoning for fishery management and conservation scallop strategy.

## Methods

### Sampling and geographic coverage

Pectinid specimens were collected between 2016 and 2019 by hand by SCUBA diving or apnea in the Magallanes Region between 50°S and 56°S in the shallow subtidal at five localities selected by specific habitat characteristics (Fig. 1): (1) Parker Island (*Reina Adelaida* Archipelago, PK) is extremely open to Pacific Ocean influence, however the sampling site was quite protected near a small island at 12 m depth on a very soft muddy bottom; (2) *Montañas* Fjord (*Última Esperanza,* FMO) is extremely protected, deep inside the fjord, on a soft muddy bottom at 15 m depth near a *Macrocystis pyrifera* forest; (3) *Ballena* Fjord (western Magellan Strait, BAL), on the moraine slope at 30 m depth on gravel and near the *Santa Ines* glacier at 15 m depth in a soft bottom; (4) *Pia* Fjord (Beagle Channel, Pia) is located inside the fjord on a short muddy platform at 25 m depth standing over a deep drop-off; and (5) Diego Ramirez Island (DR) is on a floor of shells and bryozoans at 152 m depth (by dredging). All specimens were immediately preserved in ethanol 95° for subsequent morphological and genetic analyses. A total of 54 individuals were collected in the five localities. Records from Jonkers^13^ plus the records of this study were entered in a dataset based on the Darwin Core standard in GBIF, adding a total of 96 records (10.15468/9nqxs7).

### Identification and morphological analyses

An exhaustive review of the morphological literature on both species was carried out to gather the best identification criteria (see Supplementary Table S1). The selected character described by Jonkers^13^ is the relation between the depth of the byssal notch (BN) and the height of the right valve (RV). To measure these characters, the right valve of all collected specimens was photographed and the valve height (VH) and the depth of the byssal notch (BND) were measured (Fig. 4A). According to Jonkers^13^, individuals with a BN equal to or greater than 4.5 mm and a BND/VH ratio of approximately 0.08 were attributed to *A. natans* (Fig. 4B–D) and those with a BN equal to or below 3.2 mm and with a BND/VH ratio of approximately 0.05 were attributed to *Z. patagonica* (Fig. 4E–H). The non-parametric Mann–Whitney U test was used to assess whether there are significant differences in the relationship (BND/VH) between species, using the R environment^52^.

### Genetic analyses

Genomic DNA was extracted from muscle tissue samples of 50 pectinids using the salting-out method^53^ or the Qiagen DNeasy Blood & Tissue kit (Qiagen, Hilden, Germany). DNA fragments of nuclear locus 28S ribosomal DNA (28S) and mitochondrial loci 12S, 16S ribosomal DNA (12S, 16S) were amplified following the conditions and using the primers listed in Supplementary Table S2. PCR products were purified and sequenced in both directions with an automatic ABI3730 XL sequencer at Macrogen Inc. (Seoul, South Korea). Sequences were checked visually and aligned using Muscle^54^, implemented in CodonCode Aligner v8.0.2. (https://www.codoncode.com/). Sequences were deposited in Genbank under accession numbers MT821797-MT821804.

### Phylogenetic analyses

Phylogenetic relationships among Pectinidae were reconstructed using Bayesian Inference (BI) and maximum likelihood (ML) on each dataset of 12S, 16S, 28S, separately (data not shown) and on a combined sequence set (12S + 16S + 28S). Given that the use of more divergent outgroup taxa may affect pectinid relationships and decrease resolution^4^, the outgroup selected here includes *Parvamussium pourtalesianum* and *Propeamussium sibogai*, two species of the family Propeamussidae that was identified as the closest sister group to Pectinidae^7^. The concatenated dataset was aligned with corresponding sequences of 90 pectinid and 2 propeamussiid species retrieved from Genbank (see Supplementary Table S3).

The substitution saturation of the concatenated data set was assessed with DAMBE^55^. The most appropriate model of evolution for each dataset was determined with the program Modeltest 3.7^56^. BI were conducted using the program MrBayes v.3.2.7^57^ using Metropolis-coupled Markov Chain Monte Carlo (MC3) priors as follows: 10,000,000 generations, two independent runs, four chains, sampling trees every 1000 generations. The burn-in period was identified by tracking the stability of highest likelihood values for each generation to determine whether they reached a plateau. ML analyses were conducted using PhyML 3.0 (http://www.atgc-montpellier.fr/phyml/;^58^); the program SMS^59^ uses the Akaike Information Criterion (AIC) to select the best model of evolution. The analysis started with a BioNJ tree. Nearest neighbour interchange (NNI) was selected as heuristic search. Statistical support for each branch was obtained by 1000 bootstraps. Both SMS and Modeltest programs selected the 'generalised time-reversible' model with a gamma distribution and a proportion of invariant sites (GTR + Γ + I^60^) as the best model of evolution of each of the three loci. 28S sequences that were lacking in Genbank for *Pseudamussium clavatum*, *P. sulcatum* and *Semipallium amicum* were replaced by missing data in the concatenated dataset. Gaps were treated as missing data.

## Supplementary Information

Supplementary Information

## References

[1] Serb JM, Shumway SE, Parsons GJ (2016). Reconciling morphological and molecular approaches in developing a phylogeny for the Pectinidae (Mollusca: Bivalvia). Developments in Aquaculture and Fisheries Science.

[2] Waller TR, Shumway SE (1991). Evolutionary relationships among commercial scallops (Mollusca: Bivalvia: Pectinidae). Scallops: Biology, Ecology and Aquaculture.

[3] Trovant B, Real LE, Parma AM, Orensanz JM, Basso NG (2019). Evolutionary relationships of the Tehuelche scallop *Aequipecten tehuelchus* (Bivalvia: Pectinidae) from the south-western Atlantic Ocean. J. Mar. Biol. Ass..

[4] Puslednik L, Serb JM (2008). Molecular phylogenetics of the Pectinidae (Mollusca: Bivalvia) and effect of increased taxon sampling and outgroup selection on tree topology. Mol. Phylogenet. Evol..

[5] Waller TR, Shumway SE, Parsons GJ (2006). Chapter 1 New phylogenies of the pectinidae (Mollusca: Bivalvia): Reconciling morphological and molecular approaches. Developments in Aquaculture and Fisheries Science.

[6] Stanley SM (1970). 125: Relation of Shell Form to Life Habits of the Bivalvia (Mollusca).

[7] Alejandrino A, Puslednik L, Serb JM (2011). Convergent and parallel evolution in life habit of the scallops (Bivalvia: Pectinidae). BMC Evol. Biol..

[8] Serb JM, Alejandrino A, Otárola-Castillo E, Adams DC (2011). Morphological convergence of shell shape in distantly related scallop species (Mollusca: Pectinidae): Shell shape convergence in scallops. Zool. J. Linn Soc..

[9] Raines BK, Poppe GT (2006). A Conchological Iconography: Family Pectinidae.

[10] Barucca M, Olmo E, Schiaparelli S, Canapa A (2004). Molecular phylogeny of the family Pectinidae (Mollusca: Bivalvia) based on mitochondrial 16S and 12S rRNA genes. Mol. Phylogenet. Evol..

[11] Saavedra C, Peña JB (2006). Phylogenetics of American scallops (Bivalvia: Pectinidae) based on partial 16S and 12S ribosomal RNA gene sequences. Mar. Biol..

[12] Osorio C (2002). Moluscos marinos en chile especies de importancia económica: guía para su identificación.

[13] Jonkers HA (2003). Late Cenozoic-Recent Pectinidae (Mollusca: Bivalvia) of the Southern Ocean and Neighbouring Regions.

[14] Rosenfeld S, Aldea C, Mansilla A, Marambio J, Ojeda J (2015). Richness, systematics, and distribution of molluscs associated with the macroalga *Gigartina skottsbergii* in the Strait of Magellan, Chile: a biogeographic affinity study. ZooKeys.

[15] Dijkstra, H. H. World-wide living scallops. http://www.scallop.nl/ (2014).

[16] Valladares C, Stotz W (1996). Crecimiento de *Chlamys patagonica* (Bivalvia: Pectinidae) en dos localidades de la Región de Magallanes, Chile. Rev. Chil. de Hist. Nat..

[17] Grau G (1959). Pectinidae of the Eastern Pacific.

[18] Brattström H, Johanssen A (1983). Ecological and regional zoogeography of the marine benthic fauna of Chile: report no. 49 of the Lund University Chile Expedition 1948–49. Sarsia.

[19] Waloszek D (1984). Variabilität, Taxonomie und Verbreitung von *Chlamys patagonica* (King and Broderip, 1832) und Anmerkungen zu weiteren Chlamys-Arten von der Südspitze Süd-Amerikas (Mollusca, Bivalvia, Pectinidae). Verhandlungen des Naturwissenschaftlichen Vereins zu Hamburg.

[20] Waloszek, D. *Chlamys patagonica* (King & Broderip, 1832), a long ‘neglected’ species from the shelf off the Patagonian coast. In *World Aquaculture Workshops, 1. An Int.l Compendium of Scallop Biology and Culture: A Tribute to James Mason. Selected papers from the 7th Int. Pectinid Workshop* 256–263 (The World Aquaculture Society, Baton Rou, 1991).

[21] Lasta M, Bremec C (1998). Zygochlamys patagonica in the Argentine sea: a new scallop fishery. [*Zygochlamys patagonica* en el Mar Argentino: una nueva pesqueria de vieira]. J. Shellfish Res..

[22] Guzmán, L. *et al. Diagnóstico del Ostión del Sur (Chlamys vitrea) en la provincia de Última Esperanza, Región de Magallanes*. Informe Final 188 (2007).

[23] Comité Científico Bentónico. *Renovación veda extractiva recursos ostión del sur y ostión patagónico y análisis de solicitud de disminución de talla mínima legal de ostión del sur, en la Región de Magallanes y Antártica Chilena. Informe técnico pesquería de ostiones (*Chlamys vitrea* y* Chlamys patagonica*) Región de Magallanes y Antártica Chilena*. http://www.subpesca.cl/portal/616/articles-101957_documento.pdf (2018).

[24] Smith EA, Lankester ER, Bell J (1902). Mollusca. Report on the Collections of Natural History Made in Antarctic Regions During the Voyage of the Southern Cross.

[25] Sherratt E, Alejandrino A, Kraemer AC, Serb JM, Adams DC (2016). Trends in the sand: directional evolution in the shell shape of recessing scallops (Bivalvia: Pectinidae): trends in the sand. Evolution.

[26] Canapa A, Barucca M, Marinelli A, Olmo E (1999). A molecular approach to the systematics of the Antarctic scallop *Adamussium colbecki*. Ital. J. Zool..

[27] Feng Y, Li Q, Kong L, Zheng X (2011). DNA barcoding and phylogenetic analysis of Pectinidae (Mollusca: Bivalvia) based on mitochondrial COI and 16S rRNA genes. Mol. Biol. Rep..

[28] Malkowsky Y, Klussmann-Kolb A (2012). Phylogeny and spatio-temporal distribution of European Pectinidae (Mollusca: Bivalvia). Syst. Biodivers..

[29] Moro G (2019). The first transcriptomic resource for the Antarctic scallop *Adamussium colbecki*. Mar. Genomics.

[30] Marwick J (1928). The Mollusca of the Chatam Islands including a generic revision of the New Zealand Pectinidae. Trans. N. Z. Inst..

[31] Quaglio F, Anelli LE, dos Santos PR, de Jesus Perinotto JA, Rocha-Campos AC (2008). Invertebrates from the Low Head Member (Polonez Cove Formation, Oligocene) at Vauréal Peak, King George Island, West Antarctica. Antarct. Sci..

[32] Quaglio F, Whittle RJ, Gaździcki A, Guimarães SM (2010). A new fossil Adamussium (Bivalvia: Pectinidae) from Antarctica. Pol. Polar Res..

[33] Berkman PA (2004). Polar emergence and the influence of increased sea-ice extent on the Cenozoic biogeography of pectinid molluscs in Antarctic coastal areas. Deep Sea Res. Part II Top. Stud. Oceanogr..

[34] Pirrie D, Jonkers H, Smellie JL, Crame JA, McArthur J (2011). Reworked late Neogene *Austrochlamys anderssoni* (Mollusca: Bivalvia) from northern James Ross Island, Antarctica. Antarct. Sci..

[35] Quilty PG, Murray-Wallace CV, Whitehead JM (2004). *Austrochlamys heardensis* (Fleming, 1957) (Bivalvia: Pectinidae) from Central Kerguelen Plateau, Indian Ocean: palaeontology and possible tectonic significance.. Antarct. Sci..

[36] Schiaparelli S, Linse K (2006). A reassessment of the distribution of the common Antarctic scallop *Adamussium colbecki* (Smith, 1902). Deep Sea Res. Part II Top. Stud. Oceanogr..

[37] King PP, Broderip WJ (1832). Description of the Cirrhipeda, Conchifera and Mollusca, in a collection formed by the officies of HMS Adventure and Beagle employed between the years 1826 and 1830 in surveying the southern coast of South America including the Straits of Magallanes and the coast of Tierra del Fuego. Zool. J..

[38] Schejter L, El Bremec CS (2012). nombre científico de la vieira patagónica. Rev. Invest. Des. Pesq..

[39] Dijkstra HH, Köhler F (2008). An annotated catalogue of Recent Pectinoidea (Mollusca, Pectinidae and Propeamussiidae) type material in the Museum of Natural History, Humboldt University, Berlin. Zool. Reihe.

[40] Philippi RA (1845). Diagnosen einiger neuen Conchylien. Archiv für Naturgeschichte.

[41] Dell KR (1964). Antarctic and subantarctic Mollusca: Amphineura, Scaphopoda and Bivalvia. Discov. Rep..

[42] Guzmán, L. & Valladares, C. *Programa de capacitación para los identificación de las dos especies de ostiones en la Región de Magallanes*. Informe Final 1–15 (1995).

[43] Reeve, L. Monograph of the genus Pecten. In *Conchologia iconica; or, illustrations of the shells of molluscous animals*, Vol. 8, 1–35 (London, 1852).

[44] Zelaya DG, Häussermann V, Försterra G (2009). Bivalvia – Bivalvos. Fauna Marina Bentónica de la Patagonia Chilena.

[45] Dell R (1971). The marine mollusca of the royal society expedition to southern Chile, 1958–1959. Rec. Dom. Mus..

[46] QGIS.org. QGIS Geographic Information System. QGIS Association. http://www.qgis.org (2021).

[47] SERNAPESCA. Anuario estadístico de pesca entre los periodos 1990–2017. Departamento de Sistemas de información y Estadística, Santiago. http://www.sernapesca.cl/informes/estadisticas (2020).

[48] Fenberg PH, Roy K (2008). Ecological and evolutionary consequences of size-selective harvesting: how much do we know?. Mol. Ecol..

[49] Acosta Jofré MS (2020). Population genetic structure and demographic history of the scallop *Argopecten purpuratus* from Peru and Northern Chile: implications for management and conservation of natural beds. Hydrobiologia.

[50] Faith DP (1992). Conservation evaluation and phylogenetic diversity. Biol. Conserv..

[51] Faith DP (2008). Phylogenetic diversity: conservation scenarios based on estimated extinction probabilities and phylogenetic risk analysis. Conserv. Biol..

[52] R Core Team. R: a language and environment for statistical computing. R Foundation for Statistical Computing, Vienna, Austria. URL http://www.R-project.org/ (2020).

[53] Aljanabi S (1997). Universal and rapid salt-extraction of high quality genomic DNA for PCR- based techniques. Nucleic Acids Res..

[54] Edgar RC (2004). MUSCLE: multiple sequence alignment with high accuracy and high throughput. Nucleic Acids Res..

[55] Xia, X. & Lemey, P. Assessing substitution saturation with DAMBE. In *The phylogenetic handbook: a practical approach to DNA and protein phylogeny*, Vol. 2, 615–630 (2009).

[56] Posada D, Crandall KA (1998). MODELTEST: testing the model of DNA substitution. Bioinformatics.

[57] Ronquist F (2012). MrBayes 3.2: efficient bayesian phylogenetic inference and model choice across a large model space. Syst. Biol..

[58] Guindon S (2010). New algorithms and methods to estimate maximum-likelihood phylogenies: assessing the performance of PhyML 3.0. Syst. Biol..

[59] Lefort V, Longueville J-E, Gascuel O (2017). SMS: smart model selection in PhyML. Mol. Biol. Evol..

[60] Tavaré S (1986). Some probabilistic and statistical problems in the analysis of DNA sequences. Lect. Math. Life Sci..

