# Supplementary material for: Unveiling the unknown phylogenetic position of the scallop Austrochlamys natans and its implications for marine stewardship in the Magallanes Province
Source: Sci Rep. 2021 Mar 31;11:7241. doi: 10.1038/s41598-021-86492-9 (PMC8012595; doi:10.1038/s41598-021-86492-9)
Supplement: Supplementary file 1 — Supplementary Information [file 41598_2021_86492_MOESM1_ESM.pdf]

Unveiling the unknown phylogenetic position of the scallop *Austrochlamys natans* and its implications for marine stewardship in the Magallanes Province

Sebastián Rosenfeld[1,2,3*] Cristian Aldea[2,4], Zambra López[1,2,3,5], Claudia S. Maturana[3], Jaime Ojeda[2,6], Francisco Bahamonde[2], Camille Detrée, Andrés Mansilla[2,3], Elie Poulin[1,3] & Karin Gérard[2,4*].

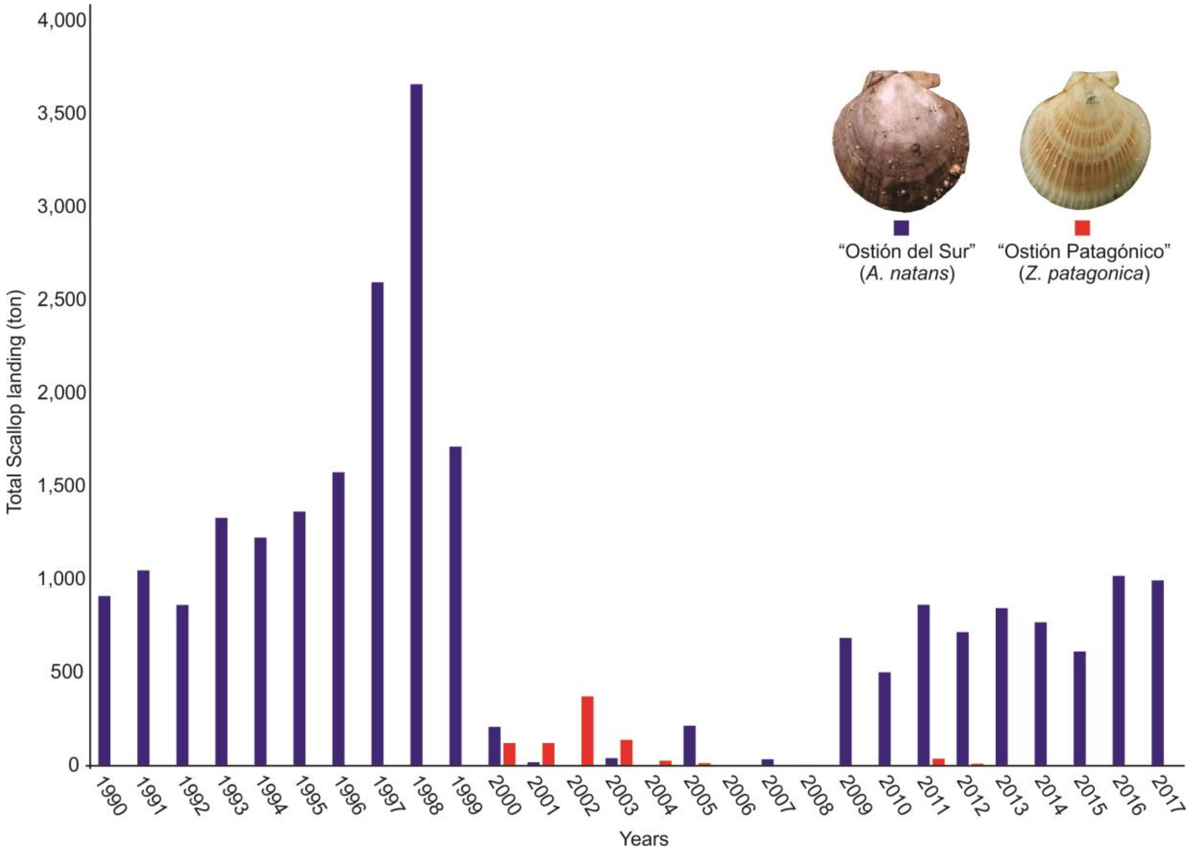

Figure S1. Fishing statistics of the scallop in the Magellan Region from 1990 to 2017.

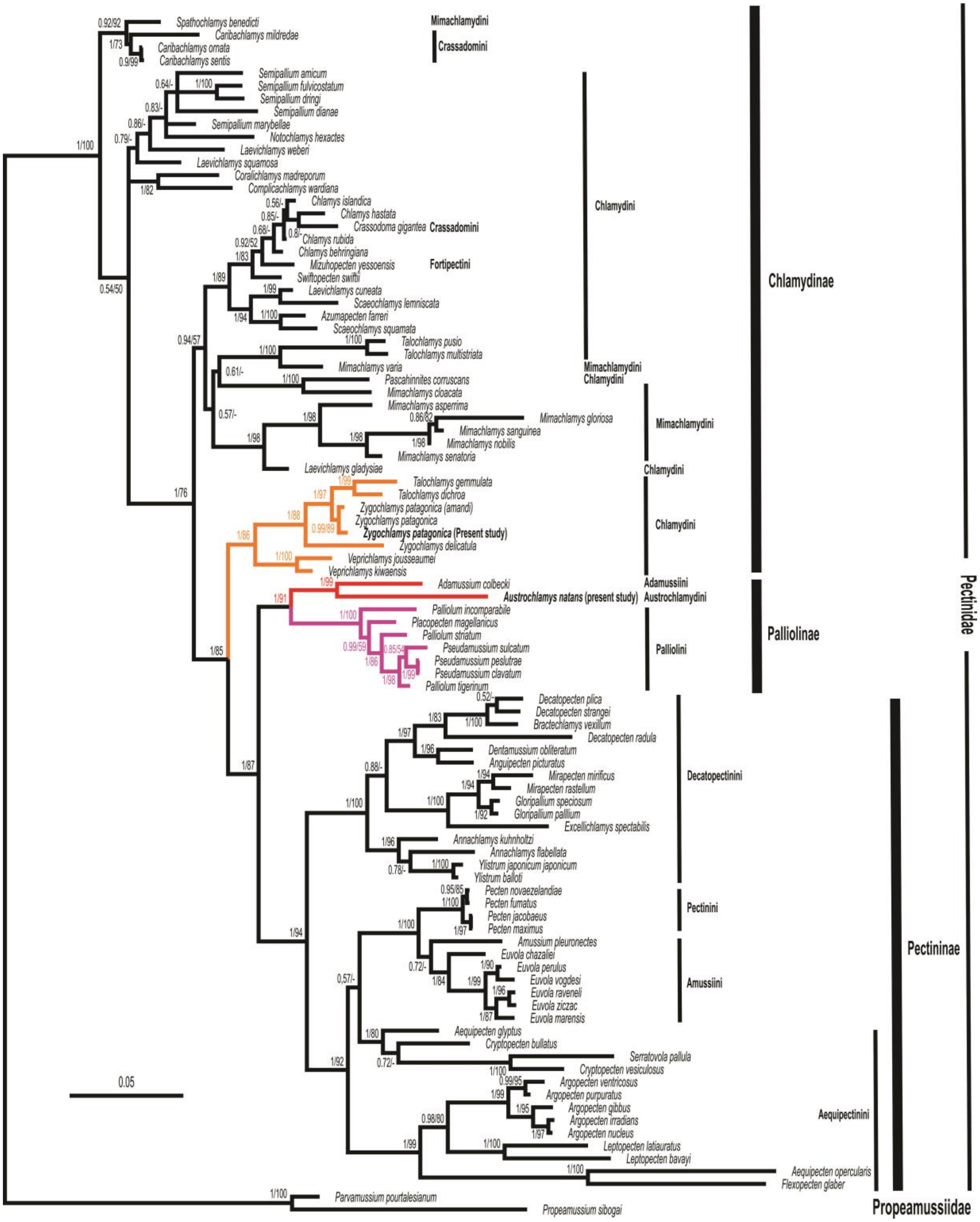

Figure S2. Phylogenetic relationships among 92 pectinid species based upon the combined dataset (12S-16S-28S) using Bayesian inference (BI) and maximum likelihood (ML) probabilistic methods. Branch support values are indicated near each node: The first number represents the portion of sampled trees in which the node was found (posterior probability, pp); the second number is the bootstrap support (bs) value >50; (-) indicates values <50.

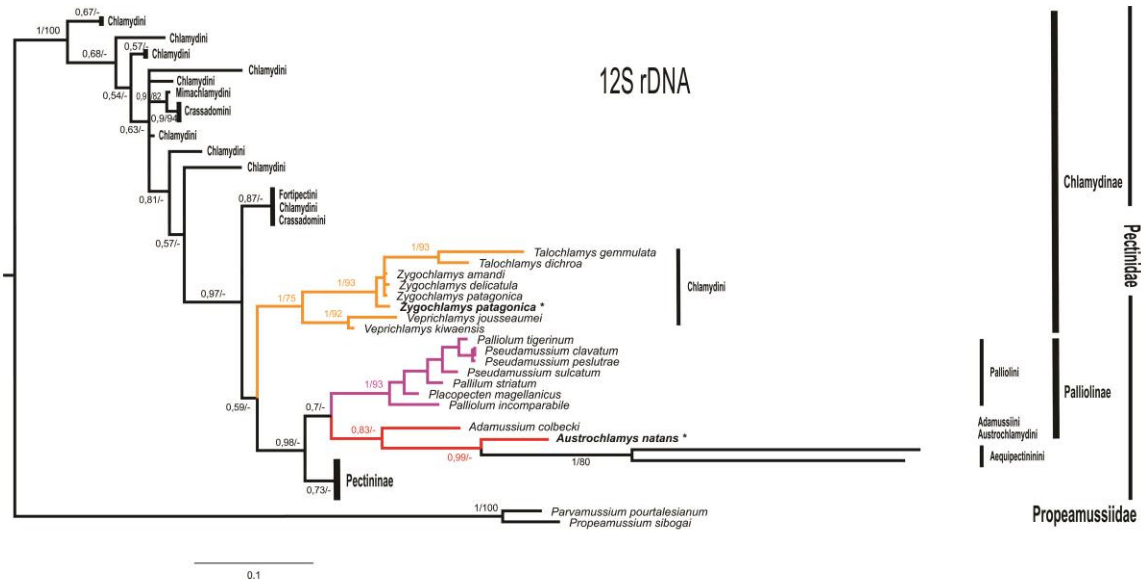

Figure S3. Phylogenetic relationships among 92 pectinid species based upon 12S rDNA sequences using Bayesian inference (BI) and maximum likelihood (ML, not shown) probabilistic methods. Branch support values are indicated near each node: The first number represents the portion of sampled trees in which the node was found (posterior probability, pp); the second number is the bootstrap support (bs) value >50; (-) indicates values <50.The (*) mark the sequences obtained in the present study.

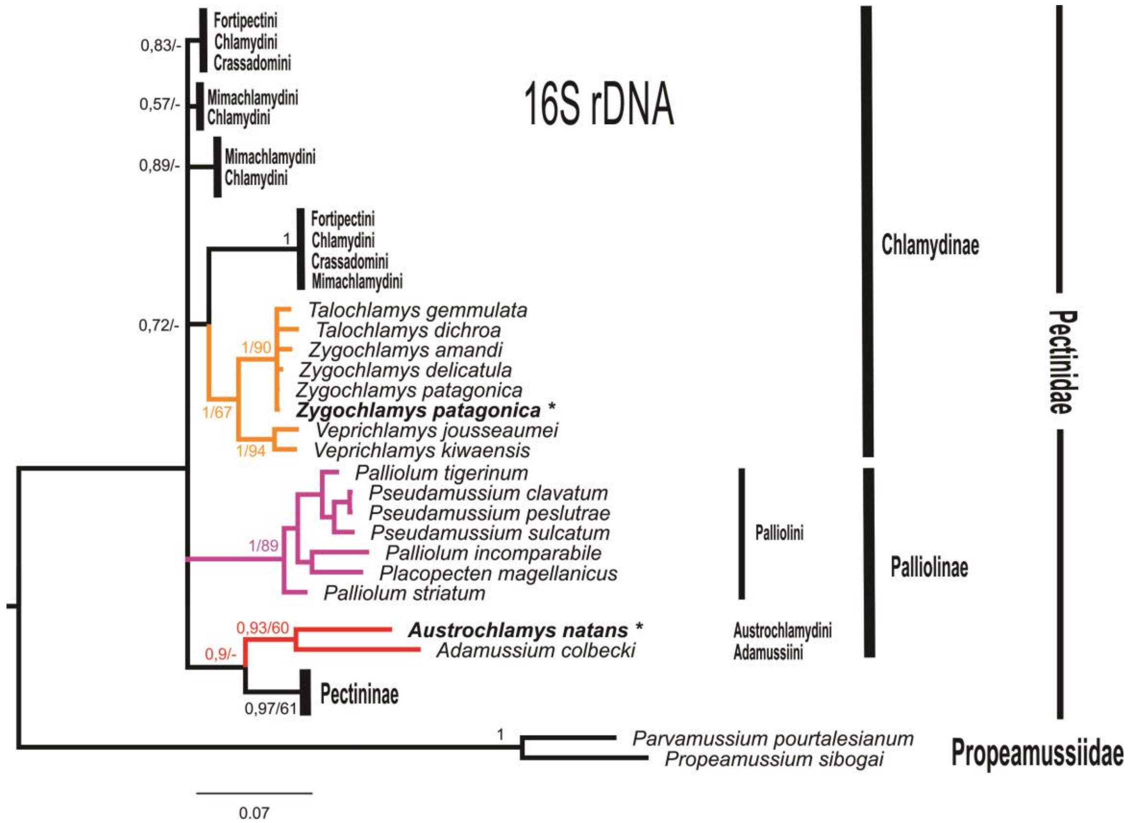

Figure S4. Phylogenetic relationships among 92 pectinid species based upon 16S rDNA sequences using Bayesian inference (BI) and maximum likelihood (ML, not shown) probabilistic methods. Branch support values are indicated near each node: The first number represents the portion of sampled trees in which the node was found (posterior probability, pp); the second number is the bootstrap support (bs) value >50; (-) indicates values <50.The (*) mark the sequences obtained in the present study.

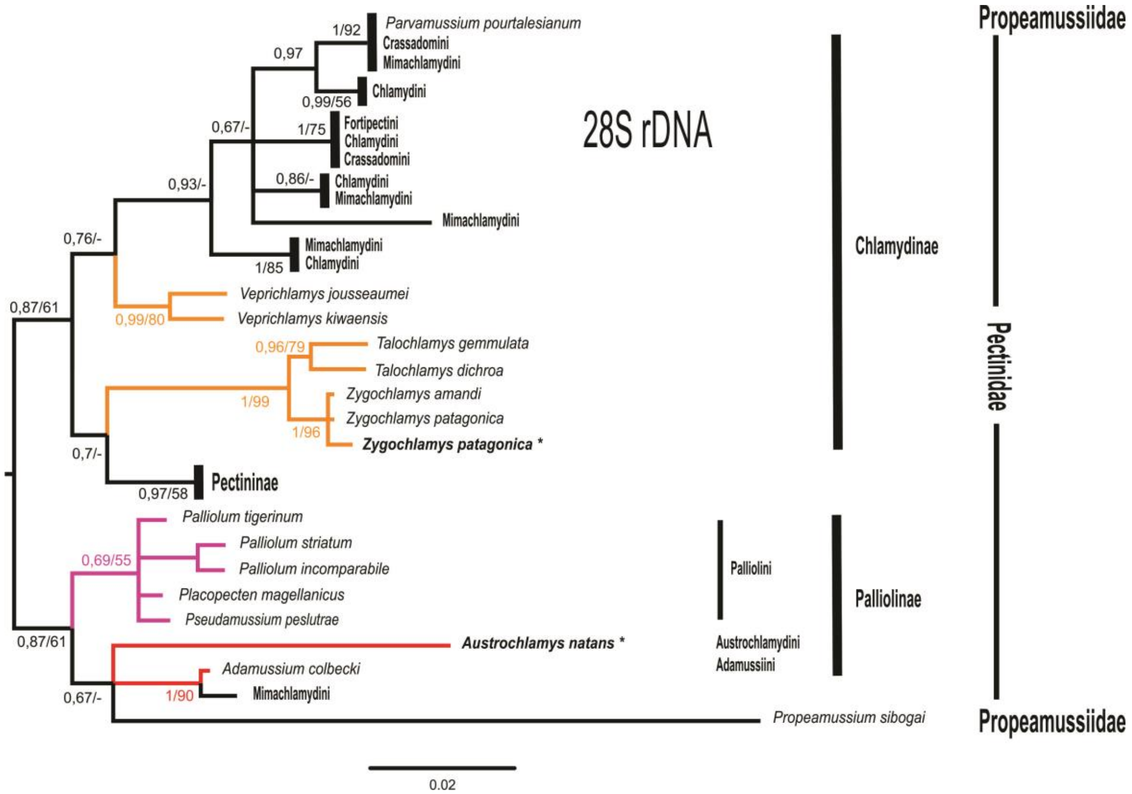

Figure S5. Phylogenetic relationships among 89 pectinid species based upon 28S rDNA sequences using Bayesian inference (BI) and maximum likelihood (ML, not shown) probabilistic methods. Branch support values are indicated near each node: The first number represents the portion of sampled trees in which the node was found (posterior probability, pp); the second number is the bootstrap support (bs) value >50; (-) indicates values <50.The (*) mark the sequences obtained in the present study.

Table S1. Historical review of the morphological and ecological on both species indicating: indicating: the typical characters used for the identification of the two species and information about the habitat (when available). LV=Left Valve and RV=Right Valve.

Nominal species	Identification characters	Habitat	Bathymetry (m)	References
Zygochlamys patagonica				
Pecten patagonicus	Brown coloring, with presence of radial ribs. Interior whitish.	-	-	[45]
Pecten australis	Ribs between 30 and 34 in adults, red coloring (LV).	-	-	[49]
Pecten lischkei	RV colors white, umbo red; LV pale orange.	-	-	[61]
Pecten rufiradiatus	Valves with 26 narrow sharp ridges transparent-white, ribs conspicuously red.	-	-	[52]
Pecten patagonicus	RV usually white color, LV red-rayed.	-	12–21	[62]
Pecten rufiradiatus	Greater height of the shell (than *P. patagonicus* King), crenulated ribs, and equal ears.	-	-	[63]
Pecten rosaceus	Rib size and number of riblets.	-	-	[64]
Pecten amandi	Rib size and number of riblets.	-	-	[65]
Chlamys amandi	LV with scaly numerous ribs, smaller size, light colored; RV yellow to red.	Stone, gravel, muddy sand, clay	5–300	[66]
Chlamys patagonica	Radiating ribs without scales or prickles, usually slightly higher than long.	-	-	[66]
Chlamys patriae	Flat valves, more oblique form.		30–190	[66]
Chlamys patagonica	RV with 23 to 28 radial ribs, pale or no color; LV white color.	Stone, Sand, mud bottoms	2–80	[17]
Chlamys (Zygochlamys) patagonica	More than 40 ribs, inflation of the shell.	Fjords of southern Chile and Atlantic coast	0–253	[50]
Chlamys patagonica	More curved and solid valves; presents between 30 to 34 main ribs.	-	-	[67]
Chlamys lischkei	Less curved, flatter valves; smaller auricules and 40 primary ribs interspersed.	-	-	[67]
Chlamys patagonica	LV with 30–32 main ribs; RV with single and bifurcated ribs that may appear as double ribs. LV white to reddish-brown or purple, pink, red and yellow coloring; RV often whitish or paler than LV.	Stone, sand, mud bottoms and kelps (fjords and Atlantic Coast)	0–200	[19]
Chlamys patagonica	Maximal shell length less than 75 mm (HV); well-marked primary and secondary radial ribs alternating regularly; RV whitish; LV white, pink, reddish-brown.	coarse and gravel, juveniles attached to adult individuals	5–100	[51]
Chlamys patagonica	Maximal shell length less than 75 mm (HV).	Stone, sand, mud bottoms and kelps (fjords and Atlantic Coast)	0–200	[16]

Nominal species	Identification characters	Habitat	Bathymetry (m)	References
Zygochlamys patagonica	Between 30 to 32 primary radial ribs; RV pink, orange or brown whit darker ribs; LV white with pink to orange ribs.	Cobbles with encrusting calcareus red algae, shelter bay	10–15	[68]
Zygochlamys patagonica	Between 22 to 42 primary radial ribs; thin, laminated, concentric lines; less volume between the shells.	Juveniles in protected bays; adults inhabit fjords with varying degrees of ice regression	15–200	[12]
Psychrochlamys patagonica	Opisthocline shell shape; symmetrical auricules and less deeper byssal notch.	Fjords and channels; Atlantic coast		[13]
Zygochlamys patagonica	Rounded oval shell with solid valves; variable outer sculpture formed by 30–40 radial ribs, which can be fine, have scales or dots.	Sand, coarse and gravel, gravel	55–285	[69]
Zygochlamys patagonica	Strong radial ribs with appearance of secondary cords; LV red, pink or orange; RV paler usually whitish.	Stone, sand, mud bottoms; juveniles associated with macroalgae	1–270	[53]
Austrochlamys natans				
Pecten vitreus	Translucent internally whitish shell, longitudinally multi-striated.	Lelps forests of *M. pyrifera*	-	[45]
Pecten natans	Oval shell, flat ribs, 25 in juveniles and 50 in adults.	-	-	[49]
Semipallium (Juxtamusium) natans	Valves moderately to rather deeply convex and frequently slightly oblique, ribs of both valves ranges from 30 to 50.	Protected coasts associated with kelp forests	SW	[17]
Chlamys vitrea	Very irregular rib pattern, 22 to 26 ribs in some specimens.	Kelps forests	-	[19]
Chlamys vitrea	Maximal shell length (HV) more than 100 mm HV; the primary and secondary ribs are weakly coloured and irregularly shaped.	Interior waters of channels and fjords; fjords with glaciers; muddy bottoms and in forests of *M. pyrifera*	SW	[51]
Chlamys vitrea	Maximal shell length (HV) more than 100 mm HV.	Kelps forests	-	[16]
Chlamys vitrea	More delicated shell; transparent when young; less distinct rounded ribs.	kelps forests in *M. pyrifera*	-	[68]
Chlamys vitrea	33 to 44 flattened primary radial ribs; with secondary striations between the primary ribs and with regular concentric lines.	-	15–200	[12]
Austrochlamys natans	Prosocline shell shape; less symmetrical auricules and much deeper byssal notch	Fjords and channels		[13]
Chlamys vitrea	Presents flattened cuadrangular radial ribs without scales.	-	-	[69]

Nominal species	Identification characters	Habitat	Bathymetry (m)	References
Austrochlamys natans	More globular, delicate shell and wider radial cords; violet coloration sometimes brownish.	-	-	53

Table S2. Amplification of ribosomal loci: Primer name, sequence and source; annealing temperature (T) and length of amplified fragment (L) in base pairs (pb) for *Zygochlamys patagonica* (*) and *Austrochlamys natans* (**).

Locus	Source	Primers	Sequences	T	L
28S rDNA	7	sc28S_70F	5’ CAGCACCGAATCCCTCAGCCTTG 3’	**50ºC**	900
		sc28S_950R	5’ TCTGGCTTCGTCCTACTCAAGCATAG 3’		
16S rDNA	69	16S-RA	5' CGCCTGTTTAKCAAAAACAT 3'	**48°C**	695
		16S-RB	5' CCGGTCTGAACTCAGATCACGT 3'		
12S rDNA	10	12-F	5’ AGACATGGATTAGATACCC 3’	**50ºC**	460*/ 420**
		12-R	5’ ACCCCTACCTTGTTACGACTT 3’		

Table S3. Genbank accession numbers of sequences used for phylogenetic reconstructions. Classification in Subfamilies and tribes followed Dijkstra[15].

**FAMILY PECTINIDAE**	12S	16S	28S
**Subfamily Pectininae**			
Decatopectini			
Bractechlamys vexillum	JF339075(71)	KP900979(72)	HM630398(7)
Anguipecten picturatus	HM630510(7)	HM630511(7)	HM630513(7)
Dentamussium obliteratum	KP300595(33)	KP300561(33)	KP300528(33)
Decatopecten strangei	HM630439(7)	JF339134(71)	HM630442(7)
Decatopecten plica	HM630435(7)	DQ640890(73)	HM630438(7)
Decatopecten radula radula	KC879129(74)	KF982788(72)	HM630494(7)
Ylistrum balloti	EU379379(4)	KC879134(74)	KC879137(74)
Ylistrum japonicum japonicum	HM622706(7)	KP900981(72)	HM622709(7)
Annachlamys flabellata	KP300578(33)	JF339137(71)	KP300515(33)
Annachlamys kuhnholtzi	KP300587(33)	JF339105(71)	KP300522(33)
Excellichlamys spectabilis	HM630457(7)	HM630458(7)	AJ307544(76)
Gloripallium pallium	EU379410(4)	AJ571609(10)	HM630525(4)
Gloripallium speciosum	HM630465(7)	HM630466(7)	AB102744(74)
Mirapecten rastellum	AJ571601(10)	AJ571613(10)	HM630456(7)
Mirapecten mirificus	AJ571600(10)	EU379455(4)	HM630540(7)
Aequipectini			
Aequipecten glyptus	EU379391(4)	EU379445(4)	HM622699(7)
Aequipecten opercularis	JQ611462(36)	EU379462(4)	HM630527(7)
Argopecten nucleus	EU379406(7)	EU379461(7)	HM630528(7)
Argopecten purpuratus	EU379417(4)	AJ972426(11)	HM630495(7)
Argopecten ventricosus	HM630407(7)	AJ972428(11)	HM630410(7)
Argopecten irradians	EU379378(4)	HQ329396(77)	AY145391(78)

Argopecten gibbus	EU379388[4]	EU379443[4]	HM622698[7]
Cryptopecten vesiculosus	HM630403[7]	HM630404[7]	HM630406[7]
Cryptopecten bullatus	KP300573[33]	KP300539[33]	KP300510[33]
Cryptopecten nux	JF339043[71]	JF339090[71]	KP300527[33]
Flexopecten glaber	JQ611466[36]	JQ611443[36]	AJ307545[76]
Leptopecten bavayi	EU379381[4]	EU379435[4]	HM540102[7]
Leptopecten latiauratus	EU379393[4]	EU379447[4]	HM622714[7]
Serratovola pallula	JF339042[71]	JF339088[71]	KP300529[33]
Amussini			
Amusium papyraceum	HM630371[7]	HM630372[7]	HM630374[7]
Amusium pleuronectes	AJ571592[10]	KC879114[74]	HM630503[7]
Euvola vogdesi	AM039768[11]	HM630388[7]	HM630390[7]
Euvola ziczac	EU379430[4]	AJ972433[4]	HM630509[7]
Euvola chazaliei	EU379382[4]	EU379436[4]	HM561999[7]
Euvola perulus	EU379413[4]	HM630517[7]	HM630514[7]
Euvola raveneli	EU379419[4]	EU379473[4]	HM630487[7]
Pectinini			
Pecten maximus	EU379400[4]	EU379454[4]	HM630545[7]
Pecten jacobaeus	JQ611477[36]	JQ611455[36]	JQ611521[36]
Pecten fumatus	HM622689[7]	JF339109[71]	HM622696[7]
Pecten novaezelandiae	JF339076[71]	JF339108[71]	HM630530[7]
**Subfamily Palliolinae**			
Palliolini			
Palliolum tigerinum	JQ611475[36]	JQ611453[36]	JQ611519[36]
Palliolum striatum	JQ611474[36]	JQ611452[36]	JQ611518[36]
Palliolum incomparabile	JQ611473[36]	JQ611450[36]	JQ611516[36]
Semipallium amicum	AJ571602[10]	AJ571614[10]	-
Semipallium marybellae	EU379399[4]	EU379453[4]	HM630546[7]
Placopecten magellanicus	FJ263638[79]	AJ972443[l]	FJ263657[79]
Pseudamussium clavatum	JF496749[80]	JF496760[80]	-
Pseudamussium sulcatum	JQ611481[36]	JF496755[80]	-
Pseudamussium peslutrae	JQ611480[36]	JQ611458[36]	FJ263659[79]
Adamussini			
Adamussium colbecki	EU379383[4]	GU227001[81]	FJ263652[79]
**Subfamily Chlamydinae**			
Chlamydini			
Azumapecten farreri	FJ263634[79]	HM622682[7]	HM622688[7]
Chlamys islandica	AJ571605[10]	FJ263646[79]	FJ263656[79]
Chlamys rubida	FJ263636[79]	FJ263645[79]	FJ263655[79]
Chlamys behringiana	FJ263632[79]	FJ263641[79]	FJ263650[79]
Chlamys hastata	FJ263639[79]	KF982789[72]	FJ263658[79]
Complicachlamys wardiana	JF339086[71]	JF339132[71]	KP300534[33]
Coralichlamys madreporarum	AJ571598[10]	AJ571608[10]	HM630548[7]
Laevichlamys squamosa	EU379426[4]	EU379480[4]	HM630443[7]
Laevichlamys weberi	KP300603[33]	KP300568[33]	KP300536[33]
Laevichlamys cuneata	HM622702[7]	HM622703[7]	HM622705[7]
Notochlamys hexactes	JF339065[71]	JF339111[71]	KP300520[33]
Pascahinnites coruscans	HM630483[7]	EU379438[4]	HM600739[7]
Scaeochlamys squamata	HM630444[7]	HM630445[7]	HM630447[7]
Scaeochlamys lemniscata	HM622715[7]	KP300554[33]	HM622718[7]
Semipallium fulvicostatum	JF339080[71]	KP300546[33]	KP300516[33]
Semipallium dringi	EU379387[4]	EU379441[4]	HM622672[7]
Semipallium dianae	HM630553[7]	HM630554[7]	HM630556[7]

Swiftopecten swiftii	KP300599(33)	KP300565(33)	KP300532(33)
Talochlamys pusio	JQ611483(36)	HM600757(7)	HM600750(7)
Talochlamys multistriata	HM630426(7)	JQ611460(36)	HM630538(7)
Talochlamys gemmulata	JF339050(71)	JF339106(71)	KP300517(33)
Talochlamys dichroa	JF339056(71)	JF339131(71)	KP300514(33)
Veprichlamys jousseaumei	HM622710(7)	HM622674(7)	HM622713(7)
Veprichlamys kiwaensis	JF339072(71)	JF339097(71)	KP300521(33)
Zygochlamys delicatula	JF339073(71)	JF339100(71)	KP300513(33)
Zygochlamys patagonica (amandi)	HM485575(7)	HM485576(7)	HM535654(7)
Zygochlamys patagonica	EU379412(4)	HM630521(7)	HM630523(7)
Mimachlamydini			
Mimachlamys varia	JQ611468(36)	JQ611446(36)	HM630415(7)
Mimachlamys cloacata	JF339052(71)	HM562001(7)	HM562003(7)
Mimachlamys asperrima	HM540080(7)	JF339125(71)	HM540087(7)
Mimachlamys gloriosa	JF339083(71)	JF339123(71)	KP300519(33)
Mimachlamys nobilis	HM630531(7)	GU119965(35)	HM630534(7)
Mimachlamys sanguinea	JF339082(71)	KF982790(72)	KP300530(33)
Mimachlamys senatoria	HM630479(7)	DQ873935(82)	HM630482(7)
Spathochlamys benedicti	HM540103(7)	HM540104(7)	HM540106(7)
Fortipectini			
Mizuhopecten yessoensis	JQ611469(36)	DQ640893(73)	HM630386(7)
Crassadomini			
Crassadoma gigantea	EU379390(4)	FJ263644(79)	FJ263654(79)
Caribachlamys mildredae	HM630541(7)	HM630542(7)	HM630544(7)
Caribachlamys ornata	HM630379(7)	HM630380(7)	HM630382(7)
Caribachlamys sentis	GU953232(8)	KX713199(83)	KX713362(83)
**FAMILY PROPEAMUSSIIDAE**			
Parvamussium pourtalesianum	EU379411(4)	EU379465(4)	HM600741(7)
Propeamussium sibogai	HM600762(7)	HM600755(7)	HM600748(7)

Additional references

61. Dunker, G. Diagnoses molluscorum quorundam novorum. *Zeitschrift für Malakozoologie* **7**, 30–32 (1850).

62. Smith, E. A. *Report on the Lamellibranchiata collected by H.M.S. Challenger during the years 1873-1876*. 1–341 (1885).

63. Melvill, J. C. & Standen, R. Notes on a collection of Marine Shells from the Lively Island, Falklands. *The Manchester Museum Owens College.Museum Handbooks* **24**, 97–105 (1898).

64. Stempell, W. Die Muscheln der Sammlung Plate. *Zool. Jahrb.* **4**, 217–250, pl. 12 (1899).

65. Hertlein, L. G. The Templeton Crocker Expedition of the California Academy of Sciences, 1932. No. 25. The Recent Pectinidae. *Proc. Calif. Acad. Sci*. **21**, 301–328 (1935).
66. Soot-Ryen, T. *Pelecypoda - Reports of the Lund University Chile Expedition, 1948-49*. 1–85 (1959).
67. Klappenbach, M. A. Notas malacologicas, II., 1. *Chlamys lischkei* (Dunker, 1850) tiene prioridad sobre *Chlamys patriae* (Doello-Jurado, 1918) (Pelecypoda). *Com. Zool. Mus. Montevideo* **10**, 1–3 (1970).
68. Reid, D.G. & Osorio C. The shallow-water marine mollusca of the Estero Elefantes and Laguna San Rafael, southern Chile. Bulletin of the British Museum (Natural History). Zoology **66**(2), 109–146 (2000).
69. Cárdenas, J., Aldea, C. & Valdovinos, C. Chilean marine mollusca of Northern Patagonia collected during the CIMAR-10 Fjords Cruise. *Gayana* **72**, 202–240 (2008).
70. Palumbi, S. R., et al. "The simple fool's guide to PCR, version 2.0." *University of Hawaii, Honolulu* 45 (1991).
71. Masters, F., Duncan, P., Shapcott A. & Stokoe, R. Unpub.
72. Marin, A., Fujimoto, T. & Arai, K. The variable 5' end of the 16S rRNA gene as a novel barcoding tool for scallops (Bivalvia, Pectinidae) *Fish. Sci.* **81**, 73-81 (2015).
73. Na-Nakorn, U., Sukmanomon, S., Yoosuk, W. & Nguyen, T.T.T. Unpub.
74. Mynhardt, G., Alejandrino, A., Puslednik, L., Corrales, J. & Serb J. M. Shell shape convergence masks biological diversity in gliding scallops: a description of a new genus *Ylistrum* n. gen. (Pectinidae) from the Indo-Pacific Ocean. *J. Molluscan Stud.* **80**, 400–411 (2014).
75. Hashimoto, A. & Matsumoto, M. Unpub.
76. Hammer, S. E. Thesis. Department of Systematic Zoology and Developmental History, Vienna University, Vienna, Austria (2001).
77. Temkin, I. Molecular Phylogeny of Pearl Oysters and their Relatives (Mollusca, Bivalvia, Pterioidea). *BMC Evol. Biol.* **10** (1), 342 (2010).
78. Passamaneck, Y. J., Schander, C. & Halanych, K. M. Investigation of molluscan phylogeny using large-subunit and small-subunit nuclear rRNA sequences *Mol. Phylogenet. Evol.* **32** (1), 25-38 (2004).

79. Puslednik, L., Pairett, A. N. & Serb, J. M. Unpub. Hybridization of two closely related and sympatric scallop (Pectinidae: Bivalvia) species, *Chlamys hastata* (Sowerby II 1842) and *C. rubida* (Hinds 1845).

80. Plazzi, F., Ceregato, A., Taviani, M. & Passamonti, M. A molecular phylogeny of bivalve mollusks: ancient radiations and divergences as revealed by mitochondrial genes. *PLoS ONE* **6** (11), E27147 (2011).

81. Heimeier, D., Lavery, S. & Sewell, M. A. Using DNA barcoding and phylogenetics to identify Antarctic invertebrate larvae: Lessons from a large scale study. *Mar Genomics* **3** (3-4), 165-177 (2010).

82. Mahidol, C., Na-Nakorn, U., Sukmanomon, S., Yoosuk, W., Taniguchi, N. & Nguyen, T.T.T. Phylogenetic relationships among nine scallop species (Bivalvia: Pectinidae) inferred from nucleotide sequences of one mitochondrial and three nuclear gene regions. *J. Shellfish Res.* **26** (1), 25-32 (2007).

83. Combosch, D. J., Collins, T. M., Glover, E. A., Graf, D. L., Harper, E. M., Healy, ,J. M., Kawauchi, G. Y., Lemer, S., McIntyre, E., Strong, E. E., Taylor, J. D., Zardus, J. D., Mikkelsen, P. M., Giribet, G. & Bieler R. A family-level Tree of Life for bivalves based on a Sanger-sequencing approach. *Mol. Phylogenet. Evol.* **107**, 91-108 (2016).
